# Hybrid peripheral nerve sheath tumors: report of five cases and detailed review of literature

**DOI:** 10.1186/s12885-017-3350-1

**Published:** 2017-05-19

**Authors:** Nasir Ud Din, Zubair Ahmad, Jamshid Abdul-Ghafar, Rashida Ahmed

**Affiliations:** 1 Department of Pathology and Laboratory Medicine, Aga Khan University Hospital, Karachi, Pakistan; 2 Department of Pathology and Laboratory Medicine, French Medical Institute for Mothers & Children (FMIC), Behind Kabul Medical University Aliabad, P.O. Box: 472, Kabul, Afghanistan

**Keywords:** Hybrid PNST, Benign, Neurofibroma, Perineurioma, Schwannoma

## Abstract

**Background:**

Hybrid peripheral nerve sheath tumors (PNSTs) have been recognized recently and were first included in the 4th edition of World Health Organization (WHO) Classification of Tumors of Soft tissue and Bone, published in 2013. These tumors show combined features of more than one type of conventional benign peripheral nerve sheath tumors. The most common combinations are those of schwannoma/perineurioma followed by combinations of neurofibroma/schwannoma and neurofibroma/perineurioma. A detailed literature review of published cases is presented.

We have discussed the types and etiology, epidemiology and sites of localization, gross and microscopic appearances and immunohistochemical features of hybrid PNSTs and association of these tumors with tumor syndromes.

**Case presentation:**

We have included five cases which were diagnosed in our department as we believe that publication of these new cases is relevant for the improved understanding of these specific tumors. Four of our five patients were males, mean age was 24 years. There was wide variation in the location of these tumors. Mean size of excised tumors was 5.5 cms in the greatest dimensions. Three out of five cases represented hybrid schwannoma/perineurioma histologically. No significant nuclear atypia, mitotic activity or necrosis seen. All five cases were completely excised. All five patients are alive and well at the time of writing with no recurrence.

**Conclusion:**

Hybrid PNSTs are distinct tumors and are usually benign. However, rare case reports have described local recurrence and at least two recent case reports have described malignant transformation in these tumors. Further studies on large number of cases are required to determine the exact pathogenetic basis of these tumors.

## Background

Hybrid peripheral nerve sheath tumors (PNSTs) are benign peripheral nerve sheath tumors, which show combined features of more than one type of benign PNSTs i.e. neurofibroma, schwannoma and perineurioma. These tumors have been recognized for some time but were only recently included officially in the 4th edition of World Health Organization (WHO) Classification of Tumors of Soft tissue and Bone and the revised 4th edition of WHO Classification of Tumors of the Central Nervous System published in 2013 and 2016 respectively [1, 2].

The most common types are combinations of Schwannoma/perineurioma, which usually occur sporadically, and neurofibroma/schwannoma, which are typically associated with neurofibromatosis (NF) type 1 or 2 or with schwannomatosis. Combinations of neurofibroma/perineurioma are rare and are usually associated with NF1 [2–5].

Those associated with NF1 carry a risk of malignant transformation to malignant peripheral nerve sheath tumors (MPNSTs) [6]. However, exact rates of recurrence and malignant transformation remain largely unknown owing to extreme rarity of these tumors [7]. Rare case reports have described local recurrence [3, 4, 8]. At least two recent case reports have described malignant transformation in hybrid PNSTs [8, 9]. Another report, documenting a recurrent hybrid schwannoma/perineurioma mentioned the presence of high cellularity and nuclear pleomorphism in both the original and recurrent tumor. The Ki 67 index was over 10 and 20% in the original and recurrent tumor respectively. The authors called that tumor a hybrid schwannoma/perineurioma with low malignant potential. Criteria for malignancy in perineurioma are not well defined. WHO mentions hypercellularity, nuclear atypia with hyperchromasia, and high mitotic rate as the criteria for malignancy [1]. Hayashi et al. [10] also used the same criteria plus Ki 67 proportion index greater than 20% to denote malignancy in perineurioma.

Hybrid PNSTs have been reported in all age groups but have been most commonly reported in young adults and so far have not demonstrated any gender predilection. They show a wide anatomic distribution and can occur anywhere in the somatic soft tissues, although an occasional case has been reported in the bone. Chow et al. recently reported a case in the femur [11]. Fingers (digits) are a common site for hybrid schwannoma/perineurioma. These tumors mostly present as painless masses localized in the dermis or subcutaneous adipose tissue [3–5]. Hybrid PNSTs rarely arise from spinal or cranial nerves [2].

On gross examination, hybrid PNSTs are usually well-circumscribed nodular lesions nodular, globoid to polypoid in configuration, with firm greyish cut surface. Most tumors range between 1 to 8 cms in size [4, 8]. Histologically, these tumors demonstrate the morphologic and immunohistochemical (IHC) features of their constituent components. Most cases described in literature have two components.

Hybrid schwannoma/perineurioma tumors are circumscribed but usually unencapsulated, have a perineurioma-like lamellar or storiform, whorling architecture but have a predominantly schwannoma like cytomorphology being composed of spindle cells with wavy, tapering nuclei, pale eosinophilic cytoplasm and indistinct cell boundaries. Degenerative changes (similar to schwannomas) may be seen [2].

Hybrid schwannomas/neurofibromas have a schwannoma like component composed of cellular Antoni A areas with nuclear palisading forming verocay bodies. Neurofibroma like areas are composed of cells with wavy elongated nuclei and scant cytoplasm, fibroblasts and and a matrix of collagen fibers and mucin positive myxoid material often arranged in a plexiform architeure [3, 5].

The rare hybrid neurofibromas/perineuriomas have areas of perineuriomatous differentiation along-with areas of plexiform neurofibroma [10].

On IHC, hybrid schwannomas/perineuriomas demonstrate S100 protein and SOX10 in the schwannomatous areas, and embryonic membrane antigen (EMA), Claudin-1 and Glucose Transporter 1 (GLUT-1) in the perineuriomatous areas. Hybrid schwannomas/neurofibromas demonstrate the S100 protein and SOX10 in the schwannomatous areas while the neurofibroma component, being composed of a polymorphic cell population, demonstrates positivity for S100, SOX10, EMA and GLUT1. In hybrid neurofibroma/perineuromas, neurofibroma component demonstrates positivity as described above for S100, SOX10, EMA, Claudin 1 and Glut 1. In perineuriomatous areas, S100 expression is not seen [3, 4, 10, 12].

Hybrid PNSTs are associated with certain tumor syndromes, and more than half of hybrid PNSTs with such associations are multiple. Over 70% patients with schwannomatosis have single or multiple hybrid neurofibroma/schwannoma tumors. Similarly, over 25% patients with Neurofibromatosis (NF) 2 and about 90% patients with NF1 have single or multiple hybrid neurofibroma/schwannoma tumors. Hybrid neurofibroma/perineurioma tumors occur most commonly in association with NF1 [5, 10].

## Case presentation

We report 5 cases of hybrid PNSTs diagnosed in our department. We searched archive files of the Department of Pathology and Clinical Laboratory, Aga Khan University Hospital for cases reported as hybrid peripheral nerve sheath tumors. The principal authors (NU and ZA) reviewed the slides of all five cases. The clinical data and follow-up obtained from medical records, hospital discharge summary and telephone calls. All available hematoxylin & eosin (H&E) and IHC stained slides were reviewed and reassessed.

**Fig. 1:**
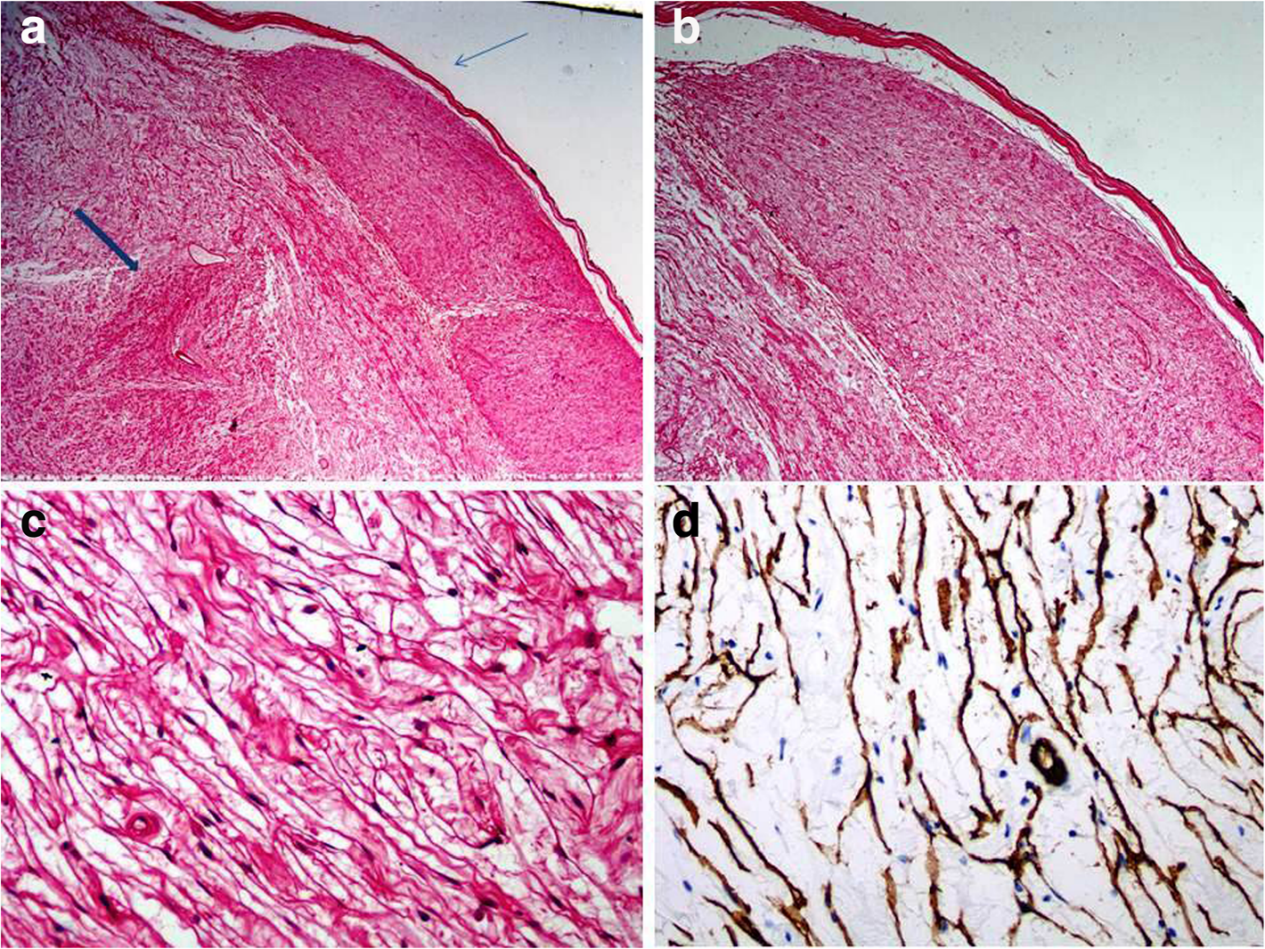
**a**, **b** Hybrid perineurioma and neurofibroma as distinct areas with perineuromatous component at top (*thin arrow*) and neurofibroma areas below it (*small thick arrow*) (H&E, 40× magnification). **c** Perineuromatous area is composed of cells with long cytoplasmic processes (H&E, 400× magnification). **d** EMA positivity in perineuromatous areas. **c**, **d** Neurofibromatous component is composed of spindle cells with wavy nuclei (100× & 400× magnifications)

**Fig. 2:**
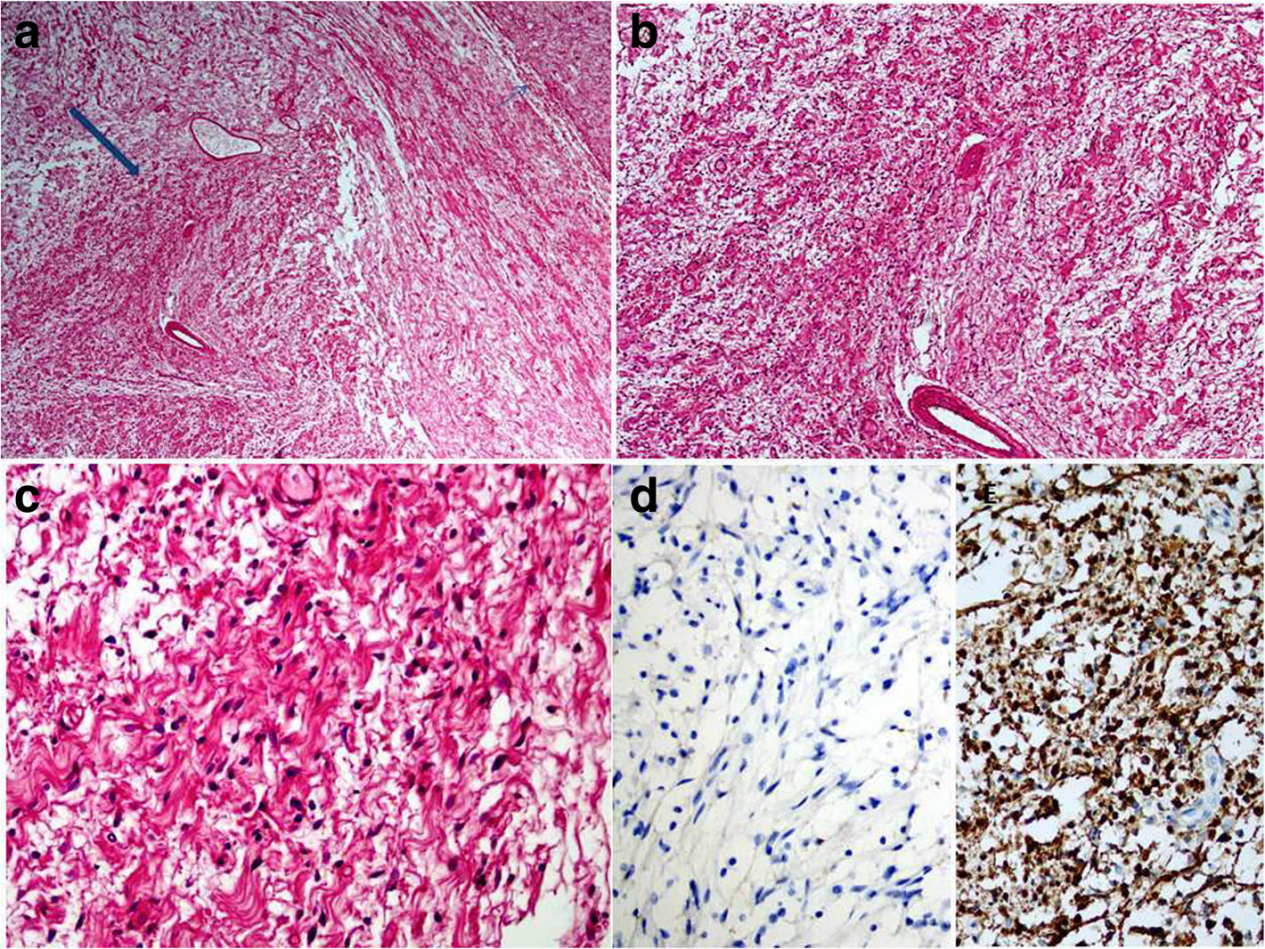
**a** Low power magnification of same case shown in Fig. 1 to highlight neurofibroma areas (*small thick arrow*). Perineuromatous focus shown at right upper corner with thin arrow. **b**, **c** Intermediate and higher magnifications of neurofibroma areas. **d**, **e** EMA negativity and S100 positivity in neurofibroma areas

**Fig. 3:**
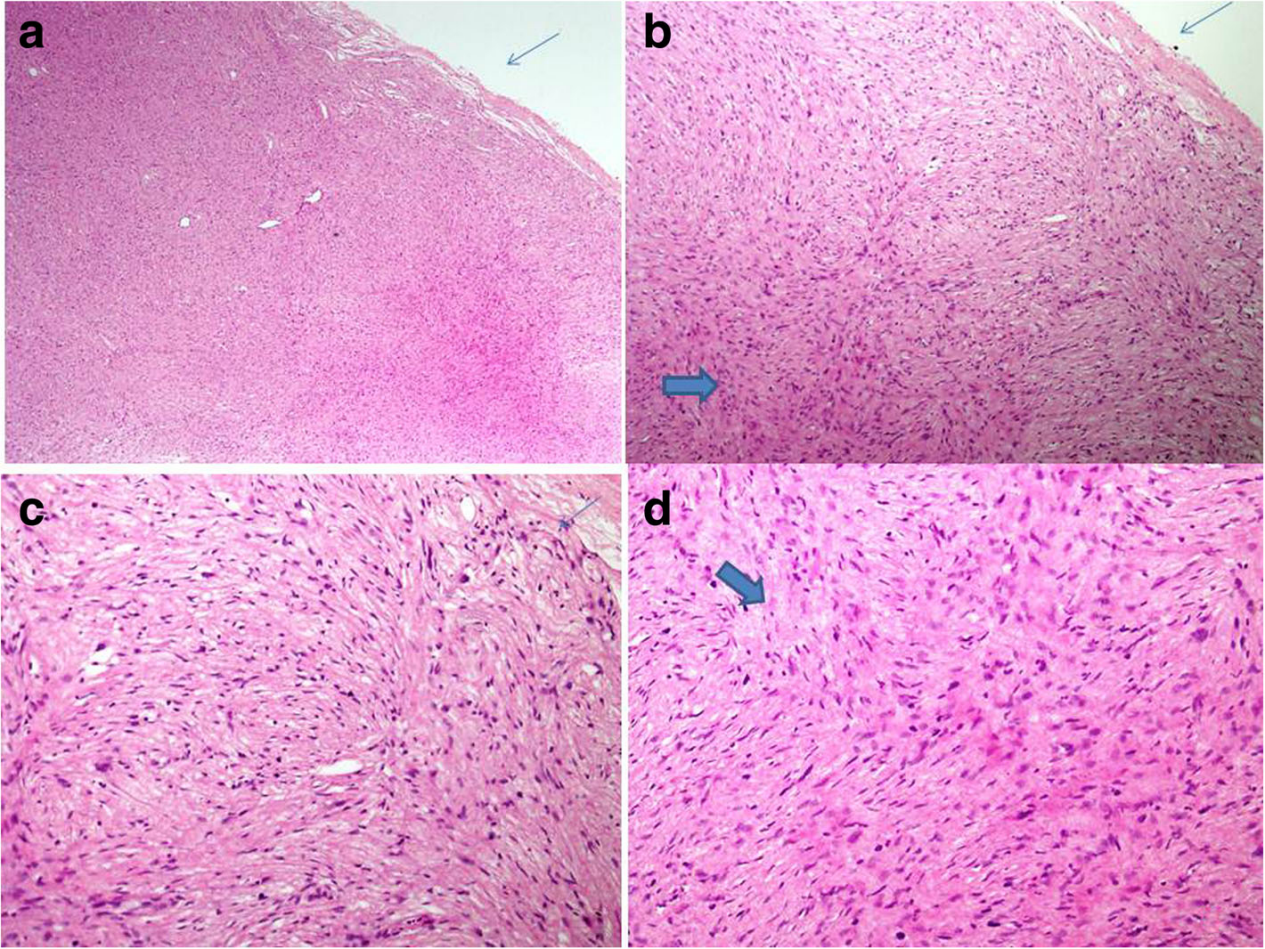
**a** to **d** Case of hybrid perineurioma and schwannoma. Perineuromatous areas shown with thin arrows at periphery and schwannoma areas shown with *thick arrows* in center

**Fig. 4:**
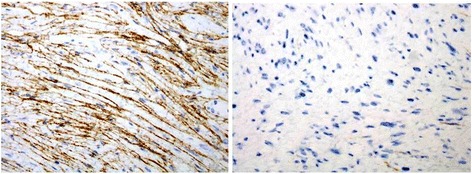
Immunoprofile of same case illustrated in Fig. 3. **a** EMA positivity in perineuromatous areas and (**b**) negativity in schwannoma areas

Out of the five cases, four were diagnosed in males and one in a female. Ages of the five patients ranged from 5 to 65 years (Mean: 24 years and Median: 12 years). Of the five cases, three patients were 12 years or younger in age. Out of all cases, one case each was located in the big toe of left foot, soft tissue of left thigh, soft tissue of right thigh, soft tissue of the neck region and retroperitoneum respectively. All patients presented with swelling or mass at the involved site. In all cases, tumors were excised and excision specimens were sent for histopathological examination. Grossly, all tumors appeared encapsulated and circumscribed; their sizes varied from 4.0 to 8.5 cm in largest dimension with a mean size of 5.5 cm. Tumors were nodular to multinodular in configuration and cut surfaces were firm, gray white to tan yellow, homogeneous to whorled to myxoidy in appearance. Histopathologically, three cases represented hybrid schwannoma/perineurioma, 1 case represented neurofibroma/perineurioma and 1 case (in the youngest patient) corresponded to schwannoma/neurofibroma (Figs. 1, 2, 3 and 4).

Microscopically, all cases showed fascicles of spindle cells with elongated, tapering, wavy to plump nuclei. Areas of nuclear palisading forming verocay bodies were also seen. Other areas demonstrated a storiform pattern. No significant mitotic activity was seen in any of the cases and no necrosis was identified. Stroma in all cases varied from loose, edematous, myxoidy to fibrillary and collagenous. Tumors in all cases were completely excised.

IHC staining of the 3 cases of hybrid schwannomas/perineuriomas showed positivity for S-100 protein in the schwannomatous areas while EMA and CD34 were positive in perineuromatous areas. The only case of neurofibroma/perineurioma also showed positivity for S100 in the neurofibromatous areas and for EMA and CD34 in the perineuromatous areas (Fig. 1d). The only case of schwannoma/neurofibroma was positive for S100 protein in both schwannomatous and neurofibromatous areas while EMA and CD34 were negative. All five cases were negative for smooth muscle actin (SMA). The clinical and pathological features of all five cases are shown in Table 1. On recent follow up, all five patients were alive and well with no evidence of residual or recurrent disease. The clinical and pathological features of all five cases are shown in Table 2.

**Table 1:** Clinical and pathological features of hybrid nerve sheath tumors in our series (*n* = 5)

Serial No	Tumor Type	Age (years)	Gender	Site	Tumor size (cm)	Positive IHC	Negative IHC stains	Year excised
1	Schwannoma/perineurioma	12	Female	Big Toe (foot)	5.0 × 4.0	S100 EMA CD34 (focal)	ASMA	2013
2	Schwannoma/perineurioma	28	Male	Thigh	8.5 × 6.0	S100 EMA CD34 (focal)	ASMA	2014
3	Schwannoma/neurofibroma	5	Male	Thigh	5.5 × 4.5	S100	EMA CD34 ASMA	2014
4	Schwannoma/perineurioma	12	Male	Neck	5.5 × 4.0	S100 EMA CD34	Desmin	2014
5	Neurofibroma/perineurioma	23	Male	Retroperitoneum	8 × 6.5	S100 EMA CD34	-	2015

**Table 2:** Comparison of current series with other published series of hybrid peripheral nerve sheath tumors

Serial No.	Study	Year published	No of cases	Tumor types	Age (Mean age/years)	M/F	Location
1	Michal et al. [8]	2004	6	Schwannoma/perineurioma	33	M1/F5	Finger & hand
2	Harder et al. [5]	2012	31	Neurofibroma/ Schwannoma	51	M18/F16	Various nerves including spinal, ulnar, axillary etc
3	Kacerovska et al. [10]	2013	5	Perineurioma/ Neurofibroma (4 cases)	41	M2/F3	Back (2), forearm, abdomen & thigh 1 case each
				Perineurioma/ Neurofibroma with malignant transformation (1 case)			
4	Yang et al. [13]	2013	10	Schwannoma/perineurioma	35	M2/F8	Subcutis of trunk (*n* = 3)
							Extremities (2), neck, nasal cavity, sigmoid colon, rectum & labia majora 1 case each
5	Requena et al.[29]	2013	9	benign cutaneous plexiform hybrid tumor of perineurioma and cellular neurothekeoma	57.6	M5/F4	Upper lip (6), lower lip (3)
6	Current study	2017	5	Schwannoma/neurofibroma (3)	16	M4/F1	Thigh (2), big toe, neck & retroperitoneum in 1 case each
				Schwannoma/neurofibroma (1)			
				Neurofibroma/perineurioma (1)			

## Discussion

Hybrid PNSTs were initially described by Feany et al. in 1998 [3] in nine patients, most of which were adults. The tumor showed components of neurofibroma and schwannoma in the same tumor. Of those cases, 2 cases were dermal and subcutaneous, while 5 were subfascial. They suggested that the presence of schwannoma and neurofibroma components together in one tumor meant that in spite of definite clinicopathologic differences, the two entities were even more closely related than was earlier thought. Zamecnik in his comment on Feany’s study called these hybrid tumors “a conceptual challenge” [13]. In 2004, Michal et al. [8] reported 6 hybrid tumors which were combinations of schwannoma and perineurioma. Out of 6 cases, five occurred on the digits and 5 were in adult females. Their cases demonstrated the classic IHC stain profile of S100+/CD34, EMA– in the schwannomatous and the S100– /CD34 and EMA+ in the perineuromatous areas. In 2005, Kazakov et al. reported 3 extradigital cases, two females and one male, all in their early fifties who had hybrid neurofibroma/perineurioma (2 cases) and hybrid schwannoma/perineurioma (1 case) which were located in the scapular area, knee and breast [14]. The same year Murarecu et al. reported a hybrid PNST that had histological and IHC features of schwannoma and neurofibroma [15]. In 2006, Emanuel et al. [16] published a case report of the first ever benign hybrid perineurioma/schwannoma outside the soft tissue located in the colon. IHC stain played a major role in differentiating this colonic tumor from a gastrointestinal stromal tumor (GIST). In 2008, Shelekhova et al. [17] reported another case of hybrid neurofibroma/perineurioma in an extradigital site. The fact that these hybrid tumors can arise in even more unusual sites, which was demonstrated by Youens et al. [18] by reporting a hybrid neurofibroma/schwannoma in the orbit of a 51-year-old female. In 2009, Hornick et al. [4] published a large series of 42 hybrid schwannoma/perineuriomas. Their cases were almost equally distributed between males and females, had a mean age of 38 years, most were subcutaneous or in the dermis, and were widely distributed in the upper and lower limbs, head and neck and trunk. One of those cases was located in the colon. Only one of their cases recurred following incomplete excision. In 2010, the report of a hybrid PNST with three components (schwannoma, neurofibroma and perineurioma, which were distinct morphologically and on IHC staining examination) in the nasopharynx demonstrated that hybrid PNSTs can have more than two components and that pathologists need to be aware of the possibility of hybrid tumors rarely occurring outside the somatic soft tissues [19].

In 2011, Hayes and O′ Sullivan reported a hybrid benign PNST in an inguinal lymph node of a 13-year-old male, demonstrating the importance of accurate diagnosis because spindle cell lesions in lymph nodes normally raise the suspicion of a metastatic tumor [20]. Similarly, in 2011, Agaimy and Michal [21] reported 2 cases of hybrid Schwannoma/perineurioma in the stomach and appendix which were both detected incidentally during surgery which were performed for suspected gastric GIST and acute appendicitis, respectively. The authors proved that such gastrointestinal hybrid PNSTs are histologically and immunohistochemically distinct from gastrointestinal schwannomas. The same year, Pusiol et al. published a paper demonstrating that the routine use of IHC stains may increase the frequency of hybrid PNSTs [22]. To our knowledge, malignant transformation of hybrid PNST was first reported by Rekhi and Jambhekar in 2011 in a young male with a mass in the right thigh who had a hybrid schwannoma/perineurioma with transformation into a malignant peripheral nerve sheath tumor (MPNST) [9]. Hybrid schwannoma/perineurioma has also been reported to occur following radiation [23]. In 2012, Park et al. [24] reported the first hybrid PNST from Korea, a hybrid perineurioma/schwannoma in the posterior mediastinum of a 53-year-old male. In 2012, Harder et al. [5] demonstrated that hybrid neurofibroma/schwannoma is a common tumor in patients with schwannomatosis and neurofibromatosis.

Also in 2012, Lang et al. [25] published a report of an even rarer occurrence of multiple, painful hybrid neurofibroma/schwannomas in the scalp, left axilla, left femoral nerve and both sciatic nerves of a 28-year-old female with no clinical features of NF type 2 or schwannomatosis and negative genetic testing for NF type 1. In 2013, a study of 10 cases of hybrid schwannoma/perineuriomas from China was published which demonstrated a marked female predominance, and a mean age of 35 years. Of 10 cases, seven were located in the subcutaneous tissues of trunk, extremities and neck while 3 cases were located in the nasal cavity, sigmoid and rectum [12]. Hybrid PNSTs when occurring in unusual sites such as gastrointestinal tract can be extremely difficult to classify and diagnostically very challenging [26]. A report by Wang et al. [27] in 2013 showed that congenital melanocytic nevus might show neural differentiation with histopathologic features of hybrid schwannoma/perineurioma. The patient was a 36-year-old male with a black tumor on his arm since birth. Also in 2013, Las Heras et al. [28] reported the first ever case of a hybrid perineurioma/schwannoma in a cranial nerve. The patient was a 24-year-old female with an internal auditory canal mass. The same year, Requena et al. [29] published a series of 9 hybrid PNSTs, all located on the lips and histologically showing distinct features of perineurioma and cellular neurothekeoma. Hayashi et al. reported multifocal intradural tumors at T11/12 and L1 in a 63-year-old-male. All were histologically consistent with hybrid schwannoma/perineurioma, had features of high cellularity, nuclear atypia and raised proliferative index which recurred five months after surgical resection as an intraneural perineurioma, showing mitotic activity and proliferative index even higher than that seen in the primary hybrid tumors [30]. Following Requena et al. [29] in 2013, Yamada et al. [31] reported a hybrid perineurioma and cellular neurothekeoma arising in the nose (unlike Requena’s nine cases, all of which were located in the lips). In 2013, which was indeed a very dynamic year for publication of reports and series of hybrid PNSTs, Kacerovska et al. [10] published a series of five cases occurring in the setting of NF Type 1. Out of those cases, one showed malignant change in the neurofibromatous component. Interestingly, three (60%) patients were members of a single family with a tragic history of various malignant neoplasms. Another hybrid perinurioma/neurofibroma in a patient with NF Type 1 was reported by Inatomi et al. in 2014 [32]. Also in 2014, Soria-Cespedes et al. [33] reported the first hybrid schwannoma/perineurioma in the pleura while Chow et al. reported a hybrid PNST in the femur, which was associated with a secondary aneurysmal bone cyst and resulted in a pathological fracture [11]. Murray et al. [34] reported a case of multiple neurofibroma/schwannoma hybrid tumors arising from the facial nerves in early 2015. In April 2015, Linos et al. reported a case of benign cutaneous biphasic hybrid tumor of perineurioma and cellular neurothekeoma (BCPHTPCN), a recently described entity that presents in the perioral area as a solitary popular lesion and microscopically demonstrates a plexiform pattern. However, their case involved the ankle as a firm, fresh colored nodule and did not show a plexiform pattern. They argued that BCPHTPCNs can grow in a non-plexiform pattern and suggested the alternate term ‘Benign cutaneous biphasic hybrid tumor of perineurioma and cellular neurothekeoma’ for these rare lesions. They also suggested that these tumors could arise outside the head and neck region [35]. In late 2015, Panda and Reena [36] reported an intraneural hybrid neurofibroma/schwannoma in the scalp of a 30-year-old male, while Taubenslag et al. [7] reported a hybrid neurofibroma/schwannoma arising from the supraorbital nerve. Also in 2015, McLaughlin et al. published the report of a hybrid PNST with three components (schwannoma/perineurioma/neurofibroma morphology), an even rarer occurrence within these extremely rare tumors. This tumor presented as a slowly enlarging, painful nodule in the upper thoracic region. It attained a size of nearly 8 cm in largest diameter over a course of five years [37].

Even today the exact pathogenetic basis of dual (or even triple) differentiation in hybrid PNSTs is poorly understood and whether such hybrid differentiation results from a clonal genetic alteration or from a localized change in the microenvironment is not known [3, 4]. Questions have even been raised about whether hybrid PNSTs really are a distinct entity or not. Very recently, Stahn et al. [38] performed a molecular analysis of 22 hybrid neurofibromas/schwannomas using immunohistochemistry, quantitative RT-PCR, array comparative genomic hybridization and cultured Schwann cells. They detected monosomy 22 (loss of chromosome 22) in 44% of their cases. Their investigations also indicated involvement of α-T-catenin/CTNNA3 in the biology of PNSTs. Published case series are summarized in Table 2.

## Conclusion

In conclusion, we are of the opinion that PNSTs with well-defined hybrid features are indeed distinct entities as demonstrated not only by their hybrid morphology, but also by their distinct hybrid IHC features. More recently, advances in molecular techniques have further documented the existence of hybrid PNSTs as distinct tumor entities. The status of hybrid PNSTs as distinct entities has been recognized by the WHO and included in the 4th edition of the WHO classification of tumors of soft tissue and bone. However, further studies are required to determine the status of hybrid PNSTs as distinct entities and to determine the exact pathogenetic basis of hybrid differentiation in PNSTs.

All five of our cases demonstrated the classical clinical, microscopic and IHC features of hybrid PNSTs. One of our cases was located in the retroperitoneum, an extremely rare location of hybrid PNSTs. None of the tumors has recurred to date and no malignant change was detected in any of the cases.

## Abbreviations

BCPHTPCN: Benign cutaneous biphasic hybrid tumor of perineurioma and cellular neurothekeoma
EMA: Epithelial membrane antigen
GIST: Gastrointestinal stromal tumor
H&E: Hematoxylin and eosin
IHC: Immunohistochemistry
MPNST: Malignant peripheral nerve sheath tumor
NF: Neurofibromatosis
PNST: Peripheral nerve sheath tumor
SMA: Smooth muscle actin
WHO: World Health Organization
